# Temporary use of leadless pacemaker as a bridge to cardiac resynchronisation therapy: a case report of fulminant myocarditis

**DOI:** 10.1093/ehjcr/ytaf316

**Published:** 2025-06-30

**Authors:** Riona Yamamoto, Naoya Kataoka, Teruhiko Imamura, Makiko Nakamura, Koichiro Kinugawa

**Affiliations:** Second Department of Internal Medicine, University of Toyama, 2630 Sugitani, Toyama 930-0194, Japan; Second Department of Internal Medicine, University of Toyama, 2630 Sugitani, Toyama 930-0194, Japan; Second Department of Internal Medicine, University of Toyama, 2630 Sugitani, Toyama 930-0194, Japan; Second Department of Internal Medicine, University of Toyama, 2630 Sugitani, Toyama 930-0194, Japan; Second Department of Internal Medicine, University of Toyama, 2630 Sugitani, Toyama 930-0194, Japan

**Keywords:** Myocarditis, Complete atrioventricular block, Leadless pacemaker, Cardiac resynchronisation therapy, Case report

## Abstract

**Background:**

Fulminant myocarditis can result in persistent severe left ventricular dysfunction and complete atrioventricular block (AVB), necessitating cardiac resynchronisation therapy (CRT). However, direct CRT implantation can be sometimes challenging in the context of intensive mechanical circulatory support and systemic steroid therapy, where the risks of procedure-related bleeding and infection are significantly heightened.

**Case summary:**

This report describes a 64-year-old woman with fulminant myocarditis, severe left ventricular dysfunction, and persistent complete AVB, who was initially managed with extracorporeal membrane oxygenation and the Impella 5.5, along with temporary pacing. Due to the high risks of infection and bleeding, we implanted a leadless pacemaker (Micra AV system) as a temporary measure prior to CRT. Following Micra AV implantation, temporary pacing was successfully removed, and signs of infection and bleeding tendency improved. CRT was subsequently implanted, and Impella 5.5 was successfully weaned.

**Discussion:**

This case underscores the potential utility of a leadless pacemaker as a bridge to CRT therapy in patients with high risks of bleeding and infection. By employing this approach, the patient’s clinical condition can be optimized, enabling the safe implantation of CRT. Further studies are warranted to validate our findings and evaluate the applicability of our novel strategy.

Learning points

- Some cases of conduction disorders, such as atrioventricular block, occurring concurrently with fulminant myocarditis, require the implantation of cardiac electronic devices.
- In cases with a higher risk of peri-procedural complications, such as bleeding related to mechanical circulatory support or infection, a leadless pacemaker may be considered as an option

## Introduction

Electrophysiological abnormalities associated with myocarditis, including atrioventricular block (AVB), occur in ∼85% of cases.^1^ Among these, advanced AVB has been reported in certain cases of myocarditis; however, once it develops, cardiogenic shock, respiratory failure, and renal failure occur more frequently.^2^ Here, we present a case of fulminant myocarditis with severely reduced left ventricular systolic function and persistent complete AVB, which required the implantation of cardiac implantable electronic devices. A leadless pacemaker was initially implanted, followed by the successful cardiac resynchronisation therapy (CRT) implantation once the patient's infectious, hemodynamics, and hemocompatibility status were stabilized. We refer to this approach as ‘Bridge to CRT using a leadless pacemaker’ and discuss its potential clinical implications.

## Summary figure

**Figure:**
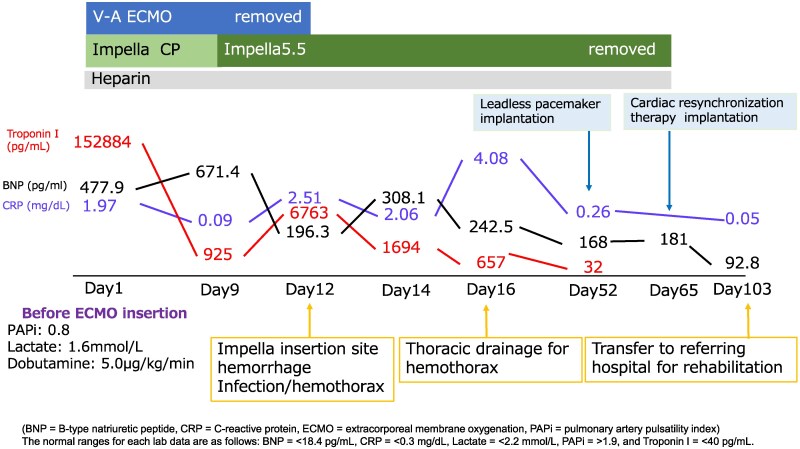


## Case presentation

### Before referral

**Figure 1:**
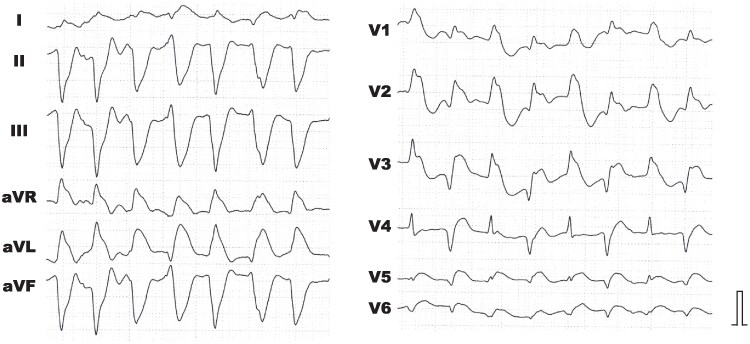
The 12-lead electrocardiogram at the initial visit. The QRS complex showed widening and ST segment elevation in leads V5 and V6. The baseline heart rhythm could not be determined.

A 64-year-old woman with no prior history except for hypertension presented with 3 days of feverish sensation, dyspnoea, and chest pain. On examination, she had a systolic blood pressure of 60 mmHg, an elevated creatine kinase of 3049 U/L (reference range: 41–153 U/L), an elevated C-reactive protein of 3.65 mg/dL (reference range: 0.00–0.14 mg/dL), and a widened QRS on the electrocardiogram (*Figure 1*). Transthoracic echocardiography revealed diffuse wall thickening and severe hypokinesis (the left ventricular ejection fraction: 35%). Coronary angiography demonstrated no significant stenosis, leading to a diagnosis of fulminant myocarditis. Following initiation of dobutamine infusion at 2 µg/kg/min and implantation of intra-aortic balloon pump (IABP) for cardiogenic shock, she was referred to our hospital for management with mechanical circulatory support.

### After referral

Despite IABP and high-dose vasopressors, she remained hemodynamically unstable with a systolic blood pressure of 70 mmHg, altered consciousness, and cold extremities. The IABP was replaced with a percutaneous left ventricular assist device (Impella CP; Abiomed, Danvers, MA) (echocardiographic images are provided in the Supplementary Materials). Following implantation of the Impella CP, the pulmonary artery pulsatility index was 0.8 (reference range ≥ 2.0), suggesting right heart failure. Furthermore, on the day of referral, complete AVB occurred, necessitating temporary pacing from the right ventricular apex. Following the initiation of right ventricular pacing, mean blood pressure further declined to below 60 mmHg. Therefore, venoarterial-extracorporeal membrane oxygenation was implemented, resulting in the establishment of ECPELLA circulation.^3^

### Following the establishment of ECPELLA circulation

The myocardial biopsy obtained from the right ventricular septum revealed severe inflammation with lymphocytic infiltration, without the presence of giant cells. Steroid pulse therapy, which consisted of 1000 mg of methylprednisolone per day for 3 days, was started. Despite treatment, haemodynamic indices remained poor, with a total bilirubin level of 2.7 mg/dL (reference range: 0.2–1.2 mg/dL) and B-type natriuretic peptide level of 460.9 pg/mL (reference range: < 18.4 pg/mL), with persistent complete AVB. On the ninth hospital day, Impella CP was upgraded to Impella 5.5 (Abiomed, Danvers, MA).

### Following the upgrade of impella

After the surgical implantation of Impella 5.5 via the right subclavian artery, uncontrolled bleeding at the insertion site necessitated re-suturing, resulting in subcutaneous haematoma and oedema across the anterior chest.

Haemodynamic stabilisation was achieved, enabling successful withdrawal of venoarterial-extracorporeal membrane oxygenation on the 12th hospital day.

On the 14th hospital day, an intrathoracic haemorrhage requiring blood transfusion occurred. Although we initially managed the Impella with a purge solution containing heparin, aiming for an activated clotting time of 160 to 180 s, continuous bleeding from the insertion site led us to reduce the target activated clotting time to below 160 s by decreasing the heparin concentration in the purge solution. However, bleeding persisted despite these adjustments, and ultimately, hemostasis was achieved through surgical suturing. Additionally, persistently elevated C-reactive protein levels suggested infection, prompting consideration of pacing lead removal. Furthermore, despite the absence of maintenance steroid therapy following pulse treatment due to the diagnosis of lymphocytic myocarditis based on the results of the myocardial biopsy, recurrent troponin I elevation (from 1820pg/mL to 6763 pg/mL; reference range: < 26.2 pg/mL) necessitated the reinitiation of prednisone at 20 mg per day.

### The implantation of a leadless pacemaker for a bridge to CRT

In light of the persistent left ventricular systolic dysfunction and complete AVB, CRT should have been optimal pacing modality. However, due to the high risk of procedure-related complications, including bleeding, infection, and haemodynamic deterioration, our team opted for leadless pacemaker implantation prior to CRT as a bridge therapy. In this strategy, we intended to wait under leadless pacing until her systemic condition would improve so that she could tolerate CRT implantation.

**Figure 2:**
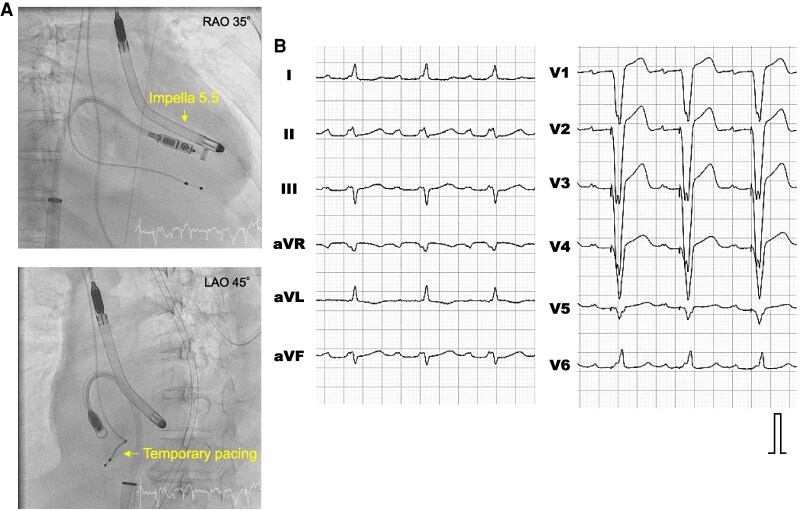
Implantation of leadless pacemaker for complete atrioventricular block. (*A*) The fluoroscopic images during leadless pacemaker implantation. RAO, right anterior oblique view; LAO, left anterior oblique view. (*B*) The 12-lead electrocardiogram recorded after the implantation of a leadless pacemaker in VDD mode.

Micra AV (Medtronic, Minneapolis, MN) in VDD mode was successfully implanted into the right ventricular septum via the right femoral vein under haemodynamic support with the Impella 5.5 on the 16th hospital day, followed by extraction of the temporary pacing lead (*Figure 2*).

### Following Micra AV implantation

Reassessment of myocardial tissue through repeat myocardial biopsy on the 30th hospital day revealed persistent lymphocytic infiltration and worsening interstitial fibrosis; therefore, our team deemed it necessary to increase the prednisone dose to 30 mg per day. Following the intensification of immunosuppressive therapy, troponin I gradually decreased, ultimately reaching 30 pg/mL. However, left ventricular systolic dysfunction persisted, with an ejection fraction of 35% and concomitant with complete AVB, making it difficult to wean the Impella.

### Bridge to CRT implantation

**Figure 3:**
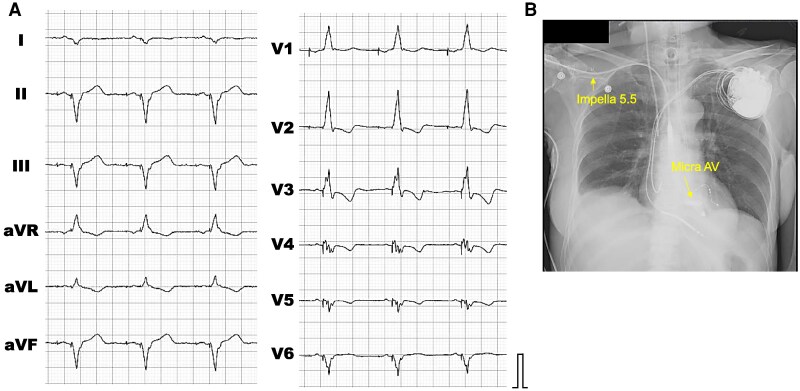
Conversion to cardiac resynchronisation therapy from leadless pacemaker under percutaneous left ventricular assist device support. (*A*) The 12-lead electrocardiogram recorded after the implantation of cardiac resynchronisation therapy. (*B*) The chest X-ray obtained 1 day after the implantation of cardiac resynchronisation therapy. The presence of the Impella 5.5 and Micra AV can be confirmed.

After confirming the resolution of the subcutaneous haematoma and subsidence of the inflammatory response, our team decided to implant CRT on the 52nd hospital day, followed by deactivation of the leadless pacemaker (*Figure 3*). Owing to haemodynamic stabilisation following CRT implantation, the Impella 5.5 was successfully weaned on the 65th hospital day. Her Troponin I levels eventually normalized, and she was transferred to another hospital for rehabilitation on the 103rd day of hospitalisation. After one month of rehabilitation, she was safely discharged to her home.

## Discussion

### Case summary

We present a case of fulminant myocarditis with severe left ventricular dysfunction and complete AVB exceeding two weeks. Signs of device-related infection necessitated the removal of the temporary pacing lead, but the patient exhibited significant bleeding tendencies and a subcutaneous haematoma. Given the higher procedural risk of CRT compared with a Micra AV system, we opted for a Micra AV implantation as a bridge to CRT.

### Atrioventricular conduction disorder in myocarditis

Complete AVB occurs in ∼12.6% of fulminant myocarditis cases, with 10%–30% recovering with steroid therapy.^4,5^ However, persistence of AVB beyond 1 week suggests irreversible Purkinje fibre damage.^6^ In the present case, prolonged myocardial inflammation may have contributed to the irreversible AVB, necessitating permanent pacemaker implantation.

### Currently recommended indications for leadless pacemakers

Leadless pacemakers eliminate vascular access challenges and reduce device-related infection risks compared with transvenous systems, making them preferable for patients on haemodialysis, with subclavian vein occlusion, or following lead extractions.^7,8^ They are also suitable for active patients. However, VDD-mode leadless pacemakers have limited atrioventricular synchronisation, and while DDD-mode devices exist, they remain unavailable in our country.^9^

### Use of a leadless pacemaker as a bridge to CRT

Temporary pacemaker lead extraction is necessary when lead infection is suspected, as demonstrated in our case. However, in such instances where bloodstream infection is suspected, the implantation of a permanent cardiac implantable electronic device is frequently deferred. The Micra AV system cannot provide conduction system or biventricular pacing, limiting its utility for long-term management of heart failure with systolic dysfunction. In this case, the Micra AV served as a temporary solution until the subcutaneous haematoma resolved and infection risk diminished. Direct CRT implantation posed higher risks due to the patient's bleeding tendency under Impella 5.5 support and increased infection susceptibility from steroid therapy. On the contrary, the Micra device has shown an infection rate of 0.002%, with a single case requiring removal due to infection being reported.^10^ This suggests superior risk reduction for infection compared with CRT implantation, which has a few percent device infection rate as a procedural complication.^11^ Thus, the leadless pacemaker was the most appropriate interim strategy to minimize complications.

Another discussion point is the use of a leadless pacemaker in the context of bleeding associated with mechanical circulatory support. In general, a purge solution containing heparin is commonly used to manage the Impella. Although our case did not utilize this approach, recent studies have reported the potential benefit of a bicarbonate-based purge solution in reducing bleeding complications.^12^ The risk of developing cardiac tamponade, which can lead to severe bleeding once it occurs, is 1.5%, higher than the 0.1%–0.3% incidence associated with transvenous leads.^13^ However, because leadless pacemakers are completely free from the issue of pocket haematoma, their superiority is expected in such bleeding-prone conditions.

Furthermore, leadless CRT may play a significant role in patients at high risk of procedure-related complications, such as bleeding or infection. Although feasibility studies have reported high complication rates, with a meta-analysis indicating rates of up to 17.8%, increased procedural proficiency is expected to mitigate these risks.^14^ Moreover, its leadless design offers a distinct advantage over transvenous leads in minimising the risk of infection and bleeding complications. Further clinical research on leadless CRT is warranted.

## Conclusion

We presented a case of complete AVB concomitant with fulminant myocarditis, in which the Micra AV system was implanted prior to CRT to reduce the risks of infection and bleeding. We propose referring to this approach as the use of a leadless pacemaker as a bridge to CRT. Such a strategy is recommended in patients, in whom direct CRT implantation is associated with high risks for procedure-related complications.

## Supplementary Material

ytaf316_Supplementary_Data

## Data Availability

The data underlying this article will be shared on reasonable request to the corresponding author.

## Lead author biography

Since 2023, Riona Yamamoto has been a Medical Staff in the Second Department of Internal Medicine, University of Toyama. In 2021-2023, she was a Junior Resident in Toyama University Hospital.

## Notes

**Consent:** The authors confirm that the patient provided informed consent for the publication of the material concerning them, in accordance with COPE guidelines.

**Funding:** None declared.
